# 

**Published:** 1888-01-28

**Authors:** 


CONTENTS.

**The Dignity of Medicine** - 293

**Words of Consolation.** Chapter LXIV.—The Sufferings of Christ - 294

**The Limits and Influences of a Nurse's Vocation.** By R. Brudenell Carter, F.R.C.S. - 295

**A Working-Class View of Our Hospitals.** By the Chairman of Workmen's Governors, Sunderland Infirmary - 296

**The National Pension Fund for Nurses and Hospital Officials** - 297

**The Doctor's Pulpit.**—A Word with the Cook. - 298

**Notes and News** - 300

**Annotations.**—Fish as "Brain Food."—A Plea for the Insane.—"Egg-Phosphate" for Over strained Nerves.—Well-paid Science - 302

**The Hospitals Association** - 303

**The Nursing Mirror** - 303

**Christmas at the Hospitals.**—By our Commissioner and Correspondents. - 304

**Rachel Challice's Vow.** By Lina Nevill, Author of "A Future on Trust," etc. - 305

**The Common Accidents of Every-day Life.**—By Robert A. P. Forrester, B.A. Dub.—Fractures and Dislocations - 307

**Medicine Men of the World.**—By the Man in the Crowd.—Chapter VI.—Savage and Civilized Practitioners - 307

**Turning over New Leaves** - 308

**Amusements with Profit for all Ages.**—For Men and Women.—Children's Corner, etc. - 310

**The average issue of "The Hospital" now exceeds 10,000 COPIES WEEKLY, and the circulation is steadily increasing**

